# Mathematical modelling of oxygenation under veno-venous ECMO configuration using either a femoral or a bicaval drainage

**DOI:** 10.1186/s40635-022-00434-x

**Published:** 2022-03-28

**Authors:** Jonathan Charbit, Elie Courvalin, Geoffrey Dagod, Pauline Deras, Thomas Laumon, Mehdi Girard, Camille Maury, Hugues Weber, Xavier Capdevila

**Affiliations:** 1 Département d’Anesthésie Réanimation Lapeyronie, Hôpital Lapeyronie, 371 Avenue du Doyen G. Giraud, 34295 Montpellier, France; 2 Critical Care Unit, Lapeyronie University Hospital, 34295 Montpellier Cedex 5, France

**Keywords:** Bicaval drainage, Oxygenation determinants, Oxygenation performance, Pulmonary shunt, Rescue therapy, Structural recirculation, Superior cava drainage, Superior cava shunt, Triple cannulation, VV-V configuration

## Abstract

**Background:**

The bicaval drainage under veno-venous extracorporeal membrane oxygenation (VV ECMO) was compared in present experimental study to the inferior caval drainage in terms of systemic oxygenation.

**Method:**

Two mathematical models were built to simulate the inferior vena cava-to-right atrium (IVC → RA) route and the bicaval drainage-to-right atrium return (IVC + SVC → RA) route using the following parameters: cardiac output (*Q*_C_), IVC flow/*Q*_C_ ratio, venous oxygen saturation, extracorporeal pump flow (*Q*_EC_), and pulmonary shunt (PULM-Shunt) to obtain pulmonary artery oxygen saturation (S_PA_O_2_) and systemic blood oxygen saturation (SaO_2_).

**Results:**

With the IVC → RA route, S_PA_O_2_ and SaO_2_ increased linearly with *Q*_EC_/*Q*_C_ until the threshold of the IVC flow/*Q*_C_ ratio, beyond which the increase in S_PA_O_2_ reached a plateau. With the IVC + SVC → RA route, S_PA_O_2_ and SaO_2_ increased linearly with *Q*_EC_/*Q*_C_ until 100% with *Q*_EC_/*Q*_C_ = 1. The difference in required *Q*_EC_/*Q*_C_ between the two routes was all the higher as SaO_2_ target or PULM-Shunt were high, and occurred all the earlier as PULM-Shunt were high. The required *Q*_EC_ between the two routes could differ from 1.0 L/min (*Q*_C_ = 5 L/min) to 1.5 L/min (*Q*_C_ = 8 L/min) for SaO_2_ target = 90%. Corresponding differences of *Q*_EC_ for SaO_2_ target = 94% were 4.7 L/min and 7.9 L/min, respectively.

**Conclusion:**

Bicaval drainage under ECMO via the IVC + SVC → RA route gave a superior systemic oxygenation performance when both *Q*_EC_/*Q*_C_ and pulmonary shunt were high. The VV-V ECMO configuration (IVC + SVC → RA route) might be an attractive rescue strategy in case of refractory hypoxaemia under VV ECMO.

**Supplementary Information:**

The online version contains supplementary material available at 10.1186/s40635-022-00434-x.

## Background

Veno-venous extracorporeal membrane oxygenation (VV-ECMO) has become the standard of care in cases of severe acute respiratory distress syndrome (ARDS) and extreme respiratory failure [1–3]. Even though the precise criteria for initiation of extracorporeal therapy are still debated, its efficacy in cases of refractory hypoxaemia has been largely demonstrated [3, 4]. However, VV-ECMO may fail to restore a satisfactory level of oxygen saturation despite a significant extracorporeal blood flow. Direct determinants of systemic oxygenation are nowadays well known: pulmonary shunt, effective extracorporeal blood flow, cardiac output, and theoretical value of mixed venous oxygen saturation (“SvO_2_”) [5, 6]. Thus, effective extracorporeal blood flow, which is directly determined by the extracorporeal settings and recirculation phenomena, is the cornerstone of blood reoxygenation under VV-ECMO. Two recirculation mechanisms have imperatively to be distinguished; the structural recirculation that mainly depends on the chosen extracorporeal route, and on the other hand the direct recirculation that depends on many technical factors (position, orientation, size, length and pattern of cannulas, extracorporeal pump flow, local impedance…) as physiological factors (cardiac output, cardiac rhythm, tricuspid regurgitations, venous impedance, intrathoracic pressure, blood volume status…). [7, 8]

Since a 20 years, the most commonly used extracorporeal route under VV-ECMO is the inferior vena cava-to-right atrium (IVC → RA) route, with two main configurations: femoro-jugular and femoro-femoral [2, 4]. This IVC → RA route is reputed simple and efficient [9]. However, despite extensive use, the IVC → RA route has two main limitations: the structural recirculation and the superior cava shunt (i.e., deoxygenated venous blood from superior vena cava [SVC] directly heading to pulmonary artery) [6–8]. These structural limits in relation to the reinfusion of reoxygenated blood in the superior vena cava (SVC) are inevitable when extracorporeal blood flow is higher than the blood flow into the IVC and cannot be avoided by a simple modification of cannulas position [6]. These two phenomena may explain extracorporeal therapy failures in cases of major pulmonary shunt.

Another extracorporeal route has been proposed to limit the phenomenon of structural recirculation and superior cava shunt: the bicaval drainage-to-right atrium return (IVC^+^SVC → RA) route [10]. The IVC^+^SVC → RA route may be performed using either a single dual-lumen cannula (e.g., Avalon Elite cannula, Getinge, Germany) or three separate single-lumen cannulas (VV-V configuration) [10–12]. Using bicaval drainage and a proximal extracorporeal return, the IVC^+^SVC → RA route should be considered as a serial configuration, which should theoretically reduce structural recirculation. More important, the superior caval shunt, which is the main factor responsible for refractory hypoxaemia under V-V ECMO, would be also reduced; this is not possible with the IVC → RA route [6]. However, despite a strong physiological rational to limit the superior caval shunt, evidences on the superiority of the IVC^+^SVC → RA route to reoxygenate systemic blood is lacking. Therefore, we wanted to compare the IVC → RA route with the IVC^+^SVC → RA route in terms of blood reoxygenation performance using a recently published ECMO mathematical model [6].

The main objective of present study was to compare the IVC → RA route (V−V configuration) to the IVC^+^SVC → RA route (VV−V configuration) using mathematical modelling (systemic oxygenation, required extracorporeal blood flow) and to identify clinical situations, where bicaval drainage might be a relevant strategy.

## Methods

### Physiological bases of the two models

Two mathematical models were built on XLSTAT 7.5.2 software (Addinsoft, New York) considering the presence of two central venous systems, the IVC and the SVC [6].

The main abbreviations used in our two models are as follows:

*Q*_C_, cardiac output.

*Q*_SVC_, blood flow in the superior vena cava.

*Q*_IVC_, blood flow in the inferior vena cava.

*k*_IVC_, proportion of cardiac output coming from the inferior vena cava.

Q_EC_, extracorporeal pump flow.

*Q*_Eff_, effective extracorporeal pump flow.

PULM-Shunt, proportion of flow crossing the pulmonary shunt.

“S_V_O_2_”, theoretical value of mixed venous blood oxygen saturation, which is the product of IVC, SVC and coronary sinus blood re-entering the heart. “S_V_O_2_” value could theoretically be assessed in absence of ECMO therapy in the pulmonary artery, but becomes unmeasurable under ECMO therapy.

S_PA_O_2_, blood oxygen saturation in the pulmonary artery.

SaO_2_, arterial blood oxygen saturation.

**Fig. 1:**
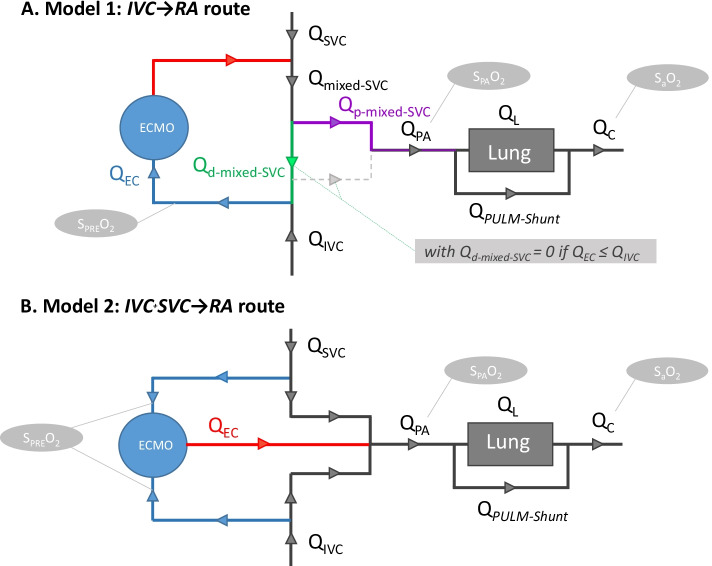
Modelling of the IVC → RA and IVC + SVC → RA routes. *IVC* inferior vena cava, *RA* right atrium, *SaO*_*2*_ arterial blood oxygen saturation, *S*_*PA*_*O*_*2*_ pulmonary arterial blood oxygen saturation, *S*_*PRE*_*O*_*2*_ blood oxygen saturation in the drainage cannula(s), *SVC* superior vena cava

The first model uses one only site of extracorporeal drainage into the IVC (the IVC → RA route), the other uses two extracorporeal drainage sites into the IVC and the SVC (the IVC^+^SVC → RA route). These two models are based on blood flows continuity and integrate blood flow and oxygen saturation for each anatomic compartment (Fig. 1). The admixture of blood produced by the ECMO reinfusion in the RA was considered to be homogeneous in these models [12, 13]. To better apprehend structural phenomena induced by these configurations, no direct recirculation was considered in the two models, leading to ideal situations for each of them. The IVC^+^SVC → RA route was, moreover, considered as a serial design without any structural recirculation when *Q*_EC_ ≤ *Q*_C_. When *Q*_EC_/*Q*_C_ > 1, *Q*_Eff_ was equal to *Q*_C_ and *Q*_EC_ could only increase due to structural recirculation.

The main equations for the two models are presented in the Additional file 1*.*

### Modelling clinical situations

The settings for four parameters (*k*_IVC_, “SvO_2_”, *Q*_C_, PULM-Shunt) were determined a priori for the two models to represent different clinical scenarios: k_IVC_ constant and arbitrary defined at 0.67 [14, 15]; “SvO_2_” constant and arbitrary defined at 60%; *Q*_C_ constant and arbitrary defined at 5 or 8 L/min; PULM-Shunt ranging from 5 to 100% in steps of 5%. For each analysis, once these clinical conditions were defined, *Q*_EC_ was gradually increased to determine SaO_2_ for each route. The *Q*_EC_ value was expressed by the *Q*_EC_/*Q*_C_ ratio in steps of 0.1 (ranging from 0 to 2).

### Study design

The consequences of PULM-Shunt on SaO_2_ were first represented according to the value of S_PA_O_2_ to better understand this pathophysiological concept under VV-ECMO, as well as the S_PA_O_2_ objectives of extracorporeal therapy, knowing that S_PA_O_2_ is the “SvO_2_” in the absence of extracorporeal therapy. A specific analysis was also performed for different target levels of SaO_2_ (90%, 94%, 98%).

Oxygenation performance of ECMO therapy was also determined for each route according the *Q*_EC_/*Q*_C_ ratio, which was proven to be a robust reflect of extracorporeal therapy (*Q*_EC_ component) on oxygenation while integrating physiological conditions and tissue needs (*Q*_C_ component). S_PA_O_2_ was thus obtained according to different “SvO_2_” levels, whereas SaO_2_ was obtained according to PULM-Shunt (“SvO_2_” constant at 60%).

Finally, the difference in required *Q*_EC_ to obtain the SaO_2_ targets (90%, 94%, 98%) between the IVC → RA and IVC^+^SVC → RA routes was calculated according to PULM-Shunt. These results were expressed using the *Q*_EC_/*Q*_C_ ratio but also as crude values of *Q*_EC_ (L/min) to better reflect clinically current situations (*Q*_C_ = 5 and 8 L/min).

## Results

### PULM-Shunt and extracorporeal therapy

**Fig. 2:**
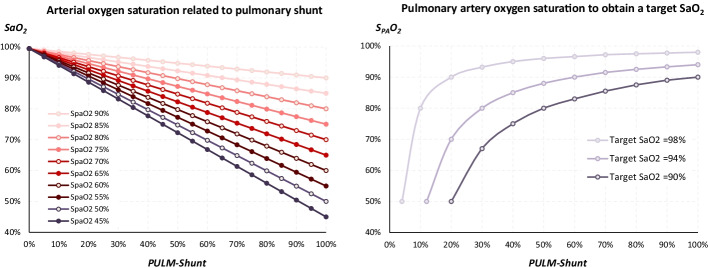
Relationship between SaO_2_ and S_PA_O_2_ according to pulmonary shunt. When lung function is optimal (PULM-Shunt=0%), SaO_2_ is equal to 100% regardless the S_PA_O_2_ value. In contrast when residual lung function is null (PULM-Shunt=100%), SaO_2_ is equal to S_PA_O_2_. Between these two clinical status, the relationship SaO_2 *_ PULM-Shunt is linear with a slope that directly depends on S_PA_O_2_. The S_PA_O_2_ value is the final consequence of ECMO therapy in term of oxygenation. *PULM-Shunt* pulmonary shunt, *SaO*_*2*_ arterial blood oxygen saturation, *S*_*PA*_*O*_*2*_ pulmonary arterial blood oxygen saturation

For a given S_PA_O_2_, SaO_2_ was linearly and inversely associated with PULM-Shunt (Fig. 2). At the two extremes: SaO_2_ = 100% when PULM-Shunt = 0% and SaO_2_ = S_PA_O_2_ when PULM-Shunt = 100%. As expected, the higher the S_PA_O_2_, the higher the SaO_2_ was for a given PULM-Shunt, highlighting the direct influence of extracorporeal therapy.

When PULM-Shunt was low, a large range of S_PA_O_2_ values allowed to obtain an SaO_2_ target of 90% or more: with a PULM-Shunt = 20%, an S_PA_O_2_ value comprised between 50 and 90% allowed to obtain an SaO_2_ target between 90 and 98% (Fig. 2B). Corresponding boundaries values of S_PA_O_2_ for a PULM-Shunt = 40% were 75% and 95%.

In contrast, when PULM-Shunt was high, only a high S_PA_O_2_, close to 90%, allowed to obtain an SaO_2_ target of 90% or more: with a PULM-Shunt = 60%, an S_PA_O_2_ value comprised between 83 and 97% allowed to obtain an SaO_2_ target between 90 and 98%. Corresponding boundaries values of S_PA_O_2_ for a PULM-Shunt = 80% were 88% and 98% (Fig. 2B).

### Oxygenation performance and extracorporeal routes

**Fig. 3:**
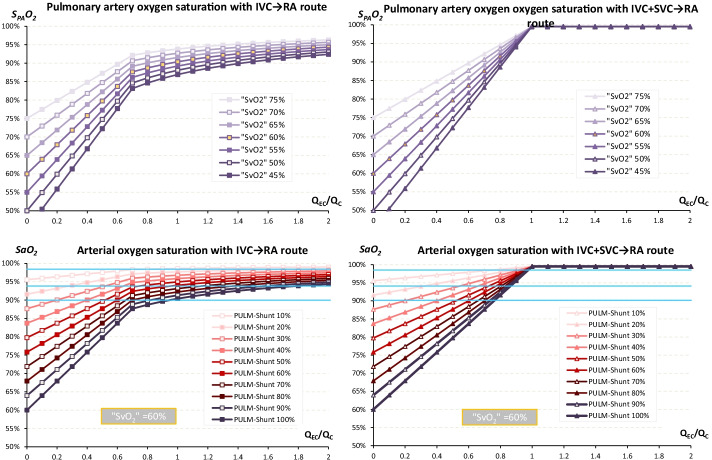
Oxygenation performance of the IVC → RA and IVC + SVC → RA routes. Beyond *Q*_EC_/*Q*_c_ = 1 using the IVC^+^SVC → RA route, *Q*_Eff_ reaches the *Q*_c_ value and cannot increase further. *Q*_EC_ increases then due to structural recirculation. *IVC* inferior vena cava, *PULM-Shunt* pulmonary shunt, *Q*_*EC*_ extracorporeal pump flow, *Q*_*Eff*_ effective extracorporeal pump flow, *Q*_*C*_ cardiac output, *RA* right atrium, *SaO*_*2*_ arterial blood oxygen saturation, *S*_*PA*_*O*_*2*_ pulmonary arterial blood oxygen saturation, *“SvO*_*2*_*”* mixed venous blood oxygen saturation, *SVC* superior vena cava

Using the IVC → RA route, S_PA_O_2_ increased linearly with *Q*_EC_/*Q*_C_ until the threshold of 0.67. Beyond this threshold, the increase in S_PA_O_2_ rise was strongly reduced and reached a plateau (Fig. 3). SaO_2_ showed the same behaviour as S_PA_O_2_, with a value directly dependent on PULM-Shunt. Despite a clinically important *Q*_EC_/*Q*_C_, SaO_2_ < 94% may occur when PULM-Shunt is significant: *Q*_EC_/*Q*_C_ = 0.6 with a PULM-Shunt > 35% for example, *Q*_EC_/*Q*_C_ = 0.8 with a PULM-Shunt > 50%, or and *Q*_EC_/*Q*_C_ = 1 with a PULM-Shunt > 60%.

In contrast, with the IVC^+^SVC → RA route, S_PA_O_2_ and SaO_2_ increased linearly with *Q*_EC_/*Q*_C_ until it reached 1, corresponding to a value of 100%. With a *Q*_EC_/*Q*_C_ > 0.86, SaO_2_ was ≥ 94% regardless of the importance of PULM-Shunt.

### *Comparison of the IVC → RA and IVC*^+^*SVC → RA routes*

**Fig. 4:**
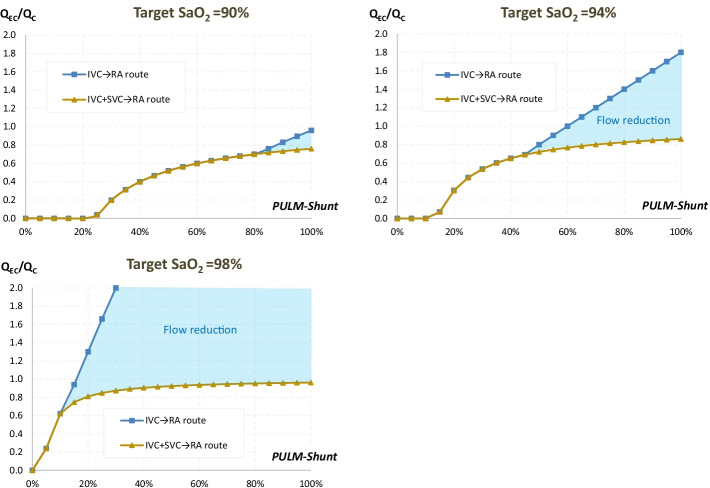
Required *Q*_EC_ to reach target SaO_2_. *IVC* inferior vena cava, *PULM-Shunt* pulmonary shunt, *Q*_*EC*_ extracorporeal pump flow, *Q*_*C*_ cardiac output, *RA* right atrium, *SaO*_*2*_ arterial blood oxygen saturation, *SVC* superior vena cava

The higher the SaO_2_ target, the more the difference in required Q_EC_ between the two routes was observed for low values of PULM-Shunt: 80% for an SaO_2_ target of 90%, 45% for an SaO_2_ target of 94%, and 12% for an SaO_2_ target of 98%. Similarly, the higher the SaO_2_ target, the higher this difference was for a given PULM-Shunt (Fig. 4).

**Fig. 5:**
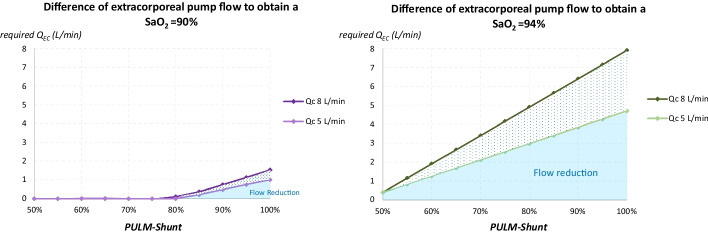
Reduction in required *Q*_EC_ from the IVC → RA route to the IVC + SVC → RA route. *IVC* inferior vena cava, *PULM-Shunt* pulmonary shunt, *Q*_*EC*_ extracorporeal pump flow, *Q*_*C*_ cardiac output, *RA* right atrium, *SaO*_*2*_ arterial blood oxygen saturation, *SVC* superior vena cava

The difference in required *Q*_EC_ between the two routes for an SaO_2_ target of 90% could reach 1.0 L/min when *Q*_C_ = 5 L/min, and 1.5 L/min when Q_C_ = 8 L/min (Fig. 5). For an SaO_2_ target of 94%, this difference in required Q_EC_ could reach 4.7 L/min and 7.9 L/min, respectively. A target SaO_2_ of 98% could not be obtained with the IVC → RA route when PULM-Shunt was 28% or more.

## Discussion

Present study compared the oxygenation performance of the IVC → RA route versus the IVC^+^SVC → RA route using mathematical ECMO models. First, our work has demonstrated that a significant pulmonary shunt imposes a high S_PA_O_2_ to maintain physiological systemic oxygenation. Second, SaO_2_ under the IVC → RA route inevitably reaches a plateau despite increasing *Q*_EC_, whereas SaO_2_ under the IVC^+^SVC → RA route increases linearly with *Q*_EC_ until 100%. The difference between these two routes occurs indeed in parallel with structural recirculation, corresponding to *Q*_EC_ > *Q*_IVC_. Third, the present study highlights that the higher the SaO_2_ target, the earlier is the difference in required *Q*_EC_/*Q*_C_ between the IVC → RA and IVC^+^SVC → RA routes, and the greater this difference for low pulmonary shunt. Our analysis has also revealed that several SaO_2_ levels cannot be obtained under the IVC → RA route when pulmonary shunt was high because of structural recirculation and superior caval shunt. Bicaval drainage could, therefore, be a part of a rescue therapy when pulmonary shunt induces refractory hypoxaemia under *the IVC → RA* route.

### Understanding the pulmonary shunt and clinical implications

The expression of pulmonary shunt as a simple percentage may appear abstract in clinical practice, especially in cases of heterogeneous ARDS or unsystematized alveolar damage. Definition of pulmonary shunt indeed is not only defined by the volume of aerated lung/alveolar collapse, but rather by the percentage of pulmonary blood circulation that will not be oxygenated through the lung; two theoretical flows into the pulmonary venous return may thus be distinguished: reoxygenated blood (SO_2_ = 100%) and deoxygenated blood (SO_2_ = S_PA_O_2_). This definition includes redistribution of the pulmonary circulation, as well as anatomic and functional pulmonary shunts [17, 18]. In clinical practice without ECMO therapy, S_PA_O_2_ and “SvO_2_” are logically equal [19]. Pulmonary shunt is simply accessible with SaO_2_, when the value of “SvO_2_” is known (SaO_2_ = 100 × (1 − PULM-Shunt) + “SvO_2_” × PULM-Shunt).

Under VV-ECMO, the “SvO_2_” becomes a theoretical value translating cellular extraction that cannot be measured. Moreover, the relationship between PULM-Shunt and SaO_2_ becomes more complex, because it is strongly influenced by the variations of S_PA_O_2_ induced by extracorporeal support (Fig. 2). Effects of VV-ECMO on oxygenation may indeed be simply summarized by an increase in S_PA_O_2_, which, combined with the PULM-Shunt, directly determines SaO_2_. For these reasons, we focused the first part of our analysis on the inter-relationship between these determinants. Thus, our work demonstrates innovatively that for a low PULM-Shunt, a large range of S_PA_O_2_ values will generate a physiological SaO_2_; in other words, a large range of *Q*_EC_ values will generate a physiological SaO_2_. In contrast, in the case of significant PULM-Shunt, only a high S_PA_O_2_ target obtained by high *Q*_EC_ will allow an acceptable SaO_2_ to be maintained. In this case, a small reduction in S_PA_O_2_ may induce a strong decrease in systemic oxygenation, highlighting the importance of extracorporeal rheological conditions in the most critical situations (Fig. 2). The present analysis allows, therefore, better understanding of the interactions between ECMO therapy and the clinical pulmonary situation. In addition, Fig. 2 shows that a high S_PA_O_2_ generated by ECMO could mask significant pulmonary impairment, which will not be visible on SaO_2_. This observation explains why the SaO2 target under ECMO therapy should not be maximal to better understand the clinical worsening and the status of alveolar recruitment.

### Structural recirculation and extracorporeal routes

Direct recirculation under VV-ECMO is well known by clinicians, unlike structural recirculation, which is less understood [7, 20, 21]. However, structural recirculation is inevitable under the IVC → RA route when *Q*_EC_ exceeds *Q*_IVC_. [6] The direct consequence is that effective *Q*_EC_, extracorporeal blood well oxygenated, becomes lower than the set *Q*_EC_. In parallel, the superior cava shunt caused by the admixture of extracorporeal reinfusion in SVC will favour hypoxaemia despite a high value of *Q*_EC_. All these mechanisms are intrinsic and inevitable limits of this route that cannot be reduced by any repositioning of cannulas. Thus, refractory hypoxaemia is possible using the IVC → RA route when the pulmonary shunt is massive despite optimized ECMO therapy (Fig. 3) [22]. This has been demonstrated by our “ideal” model that does not integrate direct recirculation. Other routes such as to right atrium-to-inferior vena cava were also proposed, but they showed a higher rate of structural recirculation [9, 12, 23]. In contrast, the IVC^+^SVC → RA route using bicaval extracorporeal drainage behaves like a serial design, without structural recirculation and with a *Q*_Eff_ close to the *Q*_EC_. The direct consequence is that the superior cava shunt may be reduced allowing systemic oxygenation to be maintained in the case of a massive PULM-Shunt (Fig. 3). Three conclusions can thus be drawn from the comparison between IVC → RA and IVC^+^SVC → RA routes. First, the difference between these two routes in terms of oxygenation is mainly observable when PULM-Shunt is severe (Fig. 4). Second, this difference of performance is higher when SaO_2_ target and/or PULM-Shunt are high. Third, the IVC^+^SVC → RA route becomes superior only when structural recirculation occurs under IVC → RA route.

### Clinical indications of bicaval drainage

Our study explores, therefore, the clinical situations, where the IVC^+^SVC → RA route may be superior to the IVC → RA route in terms of blood reoxygenation. The performances of these two routes may be considered as comparable in the absence of structural recirculation (i.e., *Q*_EC_ < *Q*_IVC_) or when PULM-Shunt is moderate. In the case of refractory hypoxaemia, the first step of management should be an increase in the *Q*_EC_/*Q*_C_ ratio, either increasing *Q*_EC_ and/or decreasing *Q*_C_ (β-blockers, sedation, etc.…) [24, 25]. However, the change of *Q*_EC_/*Q*_C_ ratio may also increase direct recirculation leading to an uncertain result. Furthermore, ELSO recommendations consider that as SaO_2_ around 85% could be an acceptable clinical value [26]. Anyway, in the presence of significant structural recirculation under IVC → RA route, the IVC^+^SVC → RA route appears to be superior in terms of systemic oxygenation. These assumptions are only valuable if direct recirculation phenomenon does not increase with the change of route. Our analysis supports thus that, in the case of refractory hypoxaemia, the IVC^+^SVC → RA route should be used when other optimization measures have failed. It is nevertheless important to understand that its added value depends on the target SaO_2_. Differences are particularly visible for high target of SaO_2_ (Fig. 4); the reduction in *Q*_EC_ using bicaval drainage may rapidly reach 1–2 L/min, which may make a real difference in clinical practice (Fig. 5). Moreover, several SaO_2_ targets will not be attainable with the IVC → RA route, because required *Q*_EC_ is too high.

### Technical considerations for the VV-V configuration

**Fig. 6:**
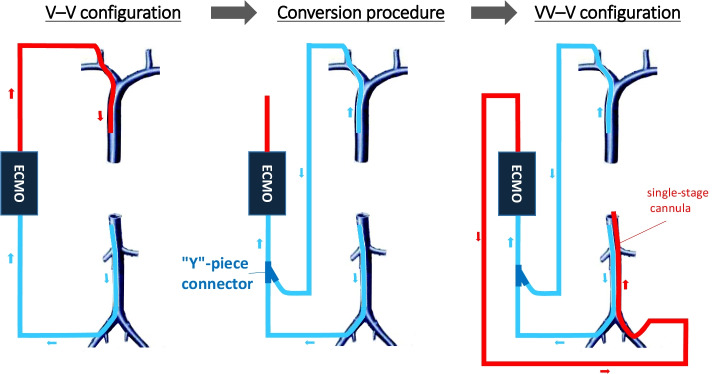
Changing the configuration from VV ECMO to VV-V ECMO. After clamping the 2 initial cannulas, tubings can be cut in conserving some length. An Y-piece connector is then positioned to reunite these 2 tubings. This Y-piece is also connected to the drainage tubing of ECMO system. Oxygenator used in V-V configuration may be conserved or changed if its performance is too altered. All tubings must be totally purged of air before connection. A complementary tubing may be necessary on return line between ECMO system and returning cannula if oxygenator is conserved. Once totally purged of air, returning cannula may be connected to the ECMO system and VV-V extracorporeal circulation may be started. In our experience, the femoral/jugular couples (29Fr–55 cm)/(22Fr–15 cm) and (27Fr–61 cm)/(20Fr–15 cm) have good balance in term of drainage, with flow percentages frequently comprised between 60%/40% and 70%/30%, respectively

Bicaval drainage can be realized as soon as implantation of ECMO using a dual-lumen cannula via the SVC and positioned across the RA [27]. However, a bicaval dual-lumen cannula imposes large diameters (≥ 27 Fr) to obtain an acceptable *Q*_EC_ and its position is reputed instable. Furthermore, direct recirculation may widely increase in cases of moderate shifting [11]. Finally, this kind of device may hardly be proposed as second step when clinical situation remains critical, because a change of configuration at this phase would be too risky. A second option could be to use a multi-staged interrupted drainage cannula upon ECMO implantation [28]. However, this configuration requires transatrial cannulation, which may be technically challenging during its placement. Furthermore, as dual-lumen cannula, repartition between superior and inferior drainage is not known and probably strongly varies with several factors (i.e., *Q*_EC_, local impedance, drainage pressure, cannula size…). Finally, a cannula change in case of critical situation does not appears without risk. A third option is the use of a triple cannulation, the VV-V ECMO configuration: two drainage cannulas (IVC and SVC) and one reinfusion cannula (RA) [10]. VV-V ECMO allows a choice of optimal and sufficient diameters for each cannula and a better precision and stability of their positioning. Superior/inferior drainage ratio may be adjusted by cannulas mobilization and external compression of the tubing to favour one or the other of the drainages to limit direct recirculation. Moreover, this attractive configuration may be proposed as a stepwise procedure if the IVC → RA route has failed (Fig. 5). For example, conversion from the femoro-jugular V-V configuration to a VV-V configuration is easy, keeping the two cannulas previously placed as drainage cannulas and repositioning a third cannula for the return of extracorporeal blood into the RA (Fig. 6). An accurate positioning in the RA and a single-stage design are, however, indispensable to limit direct recirculation. In addition to the reduction in the superior cava shunt and optimization of the *Q*_Eff_, this configuration would also allow improvement in the impedance of venous blood drainage and maximal pump flow [10]. We strongly believe that this sequenced strategy of extracorporeal respiratory support is the most appropriate way to adapt to the clinical needs of patients and their delayed worsening; a gradual response when necessary! However, prospective studies must be done to prove the safety and efficacy of this kind of strategy.

### Potential limitations of the present study

First, direct recirculation was not considered in our analysis. A bad positioning of a cannula may indeed significantly increase recirculation phenomena, especially under the IVC^+^SVC → RA route or when *Q*_EC_ is high. Furthermore, the heart cycle and tricuspid valve closure may reinforce these phenomena. Thus, behaviour of VV-V configuration, more at risk of direct recirculation and more dependent from cannula positioning, could be less efficient in term of oxygenation than the ideal model used in our works. However, it seems to us essential to understand the differences in structural recirculation according to different extracorporeal routes to identify the limitations and disadvantages of each ECMO configuration. Second, our IVC^+^SVC → RA model was built as previously detailed without any structural recirculation when *Q*_EC_ ≤ *Q*_C_. This simulates obviously an ideal situation that in clinical conditions depends on many parameters, such as position of each cannulas, theirs patterns, their diameters, local impedances, and thoracic or abdominal pressures. In clinical practice, a structural recirculation may exist under IVC^+^SVC → RA route, which logically alters performance of this configuration. Third, our model did not consider the influence of dissolved oxygen in the reoxygenated blood. A strong increase in the partial pressure of oxygen (e.g., oxygen fraction crossing the oxygenator close to 100%) may increase the mass of oxygen transferred into the extracorporeal circulation by increasing the mass of dissolved oxygen. However, we assume that this analysis bias exists similarly for each route and has a modest influence on the SaO_2_ [29, 30]. Anyway, to better understand structural recirculation phenomena, it appears more relevant to apprehend extracorporeal oxygenation determinants without mechanisms of solubility of gases. Fourth, pulmonary shunt may vary during the respiratory cycle or by a change in the airway pressure level by modifying West’s lung zones [31]. Many clinical factors influencing the pulmonary shunt could indeed be integrated into the model. However, we believe that this simplified approach offers a global understanding of the clinical situation and balance between lung and extracorporeal oxygenation, which is the central question during VV-ECMO management.

## Conclusion

Our mathematical modelling has allowed us to compare the differences between the IVC → RA and IVC^+^SVC → RA routes in terms of oxygenation performance. The usual IVC → RA route generates inevitably a structural recirculation and a superior cava shunt, which may lead to refractory hypoxaemia in extreme cases. The IVC^+^SVC → RA route would not suffer from these structural limitations. The present study suggests, therefore, that the VV-V ECMO configuration might be an attractive therapeutic option when VV-ECMO does not allow sufficient oxygenation, provided that the configuration change is not associated with a significant increase of direct recirculation.

## Supplementary Information

**Additional file 1.** Mathematical Basis of Model 1: the IVC→RA Route. Mathematical basis of Model 2: the IVC+SVC→RA Route.

## Data Availability

The data sets used and analyzed are during the current study are available from the corresponding author on reasonable request.
