# Supplementary material for: Mathematical modelling of oxygenation under veno-venous ECMO configuration using either a femoral or a bicaval drainage
Source: Intensive Care Med Exp. 2022 Mar 28;10:10. doi: 10.1186/s40635-022-00434-x (PMC8960524; doi:10.1186/s40635-022-00434-x)
Supplement: Supplementary file 1 — Additional file 1. Mathematical Basis of Model 1: the IVC→RA Route. Mathematical basis of Model 2: the IVC+SVC→RA Route. [file 40635_2022_434_MOESM1_ESM.docx]

# Mathematical modelling of oxygenation under veno-venous ECMO configuration using either a femoral or a bicaval drainage. **Additional Material**

## **Mathematical Basis of Model 1: the IVC→RA Route**

The mathematical model of the IVC→RA route is based on the presence of two central venous returns and the positioning of ECMO cannulas. Because the proportion of IVC/SVC flows may differ between patients and according to many parameters, the present model has integrated the k_IVC_ ratio as a setting parameter, representing the proportion of cardiac output (Q_C_) coming from the IVC (Q_IVC_):

Q_IVC_ = k_IVC_ × Q_C_ and Q_SVC_ = (1 − k_IVC_) × Q_C_ (1)

Because the draining cannula is positioned in the IVC, the model is then double and depends on the values of Q_EC_ and Q_IVC_ as follows:

1. If Q_EC_ ≤ Q_IVC_, the desaturated blood that is aspired by the femoral drainage cannula comes exclusively from the IVC (Q_d-IVC_) (Figure 1A):

Q_EC_ = Q_d-IVC_ (2)

2. In contrast, if Q_EC_ > Q_IVC_, some of the blood drained by the femoral drainage cannula comes logically from the SVC via the right atrium (Figure 1B). This blood from the SVC (Q_mixed-SVC_) is an admixture of the SVC drainage system (Q_SVC_) and reoxygenated blood from the returning cannula (Q_EC_):

Q_mixed-SVC_ = Q_SVC_ + Q_EC_ = (1 − k_IVC_) × Q_C_ + Q_EC_ (3)

The extracorporeal flow ratio in the SVC (EFR_SVC_) was thus determined following Equation (1), which represents the proportion of reinfused EC circulation in the mixed-SVC blood:

EFR_SVC_ = Q_EC_/Q_mixed-SVC_ = Q_EC_/(Q_SVC_ + Q_EC_) = Q_EC_/(Q_EC_ + (1 − k_IVC_) × Q_C_) (4)

The blood drained from the femoral drainage cannula is therefore the sum of three flows coming from the IVC (Q_d-IVC_), SVC (Q_d-SVC_), and reinfused EC circulation (Q_d-EC_):

Q_EC_ = Q_d-IVC_ + Q_d-mixed-SVC_ = Q _d-IVC_ + Q_d-SVC_ + Q_d-EC_ (5)

The recirculation coefficient (R) was also determined using the flow ratio following the previous equations:

R = Q_d-EC_/Q_EC_ = EFR_SVC_ × (Q_EC_ − Q_IVC_)/Q_EC_ with R = 0 if Q_EC_ ≤ Q_IVC_

R = (Q_EC_ − k_IVC_ × Q_C_)/(Q_EC_ + (1 − k_IVC_) × Q_C_) (6)

The effective extracorporeal pump flow (Q_Eff_) was defined to the extracorporeal flow of oxygenated blood reinfused in the pulmonary circulation as a serial configuration.^15,20^ On the other hand, Q_Eff_ also corresponds as the flow of desaturated venous blood crossing the ECMO system. Q_Eff_ was therefore calculated using Equations (5) and (6):

Q_Eff_ = Q_d-IVC_ + Q_d-SVC_

Q_Eff_ = (1 − R) × Q_EC_ and Q_Eff_/Q_EC_ = (1 − R) (7)

Thus, its calculation in our model depends on the values of Q_EC_ and Q_IVC_:

1. If Q_EC_ ≤ Q_IVC_, then Q_Eff_ = Q_d-IVC_ = Q_EC_ with R = 0

2. If Q_EC_ > Q_IVC_, Q_mixed-SVC_ divides into two parts: one part is drained by the drainage cannula towards the ECMO system (Q_d-mixed-SVC_); the other part goes towards the pulmonary circulation (Q_p-mixed-SVC_). Therefore, considering that Q_d-IVC_ = Q_IVC_, the Q_p-mixed-SVC_ is equal to Q_PA_ and Q_C_; therefore

Q_Eff_ = EFR_SVC_ × Q_p-mixed-SVC_ = EFR_SVC_ × Q_C_ (8)

and from Equations (4) and (8)

Q_Eff_/Q_C_ = EFR_SVC_ = (Q_EC_/Q_C_)/(Q_EC_/Q_C_ + 1 − k_IVC_) (9)

The intrapulmonary shunt (PULM-Shunt) was defined as the proportion of flow that is not oxygenated across the functional alveoli (Q_S-PULM_):

PULM-Shunt = Q_S-PULM_/Q_C_ and Q_S-PULM_ = PULM-Shunt × Q_C_ (10)

To determine oxygen blood saturations, the “SvO_2_” value (theoretical value of mixed venous oxygen saturation from tissues) was a setting parameter in our model. The oxygen blood saturation exiting from the ECMO system (S_POST_O_2_) or functional alveoli of the lung was arbitrarily determined at 99.5%. The oxygen saturation in the pulmonary artery (S_PA_O_2_) was then calculated by considering Q_Eff_ and Q_Eff_/Q_C_ and Equations (7) and (9):

S_PA_O_2_ = 99.5% × Q_Eff_/Q_C_ + “SvO_2_” × (1 − Q_Eff_/Q_C_) (11)

S_PA_O_2_ = 99.5% × (1 − R) × Q_EC_/Q_C_ + “SvO_2_” × (Q_C_ − (1 − R) × Q_EC_)/Q_C_ (12)

Finally, systemic oxygen saturation (SaO_2_) was calculated by considering the S_PA_O_2_ and intrapulmonary shunt (PULM-Shunt) from Equations (10) and (15):

SaO_2_ = 99.5% × (1 – PULM-Shunt) + S_PA_O_2_ × PULM-Shunt (13)

## **Mathematical basis of Model 2: the IVC+SVC→RA Route**

The mathematical model of the IVC+SVC→RA route is based on progressive and proportional drainage of the two venous returns. Thus*,* extracorporeal blood comes from the IVC (Q_d-IVC_) and SVC (Q_d-SVC_):

Q_EC_ = Q_d-IVC_ + Q_d-SVC_ with Q_EC_ ≤ Q_C_ (14)

To determine oxygen blood saturations, the “SvO_2_” value (theoretical value of mixed venous oxygen saturation from tissues) was a setting parameter in our model. The oxygen blood saturation exiting from the ECMO system (S_POST_O_2_) or functional alveoli of the lung was arbitrarily determined at 99.5%.

The oxygen saturation in the pulmonary artery (S_PA_O_2_) was calculated as follows:

S_PA_O_2_ = 99.5% × Q_EC_/Q_C_ + “SvO_2_” × (1 − Q_EC_/Q_C_) with Q_EC_ < Q_C_ (15)

The systemic oxygen saturation (SaO_2_) was calculated as follows:

SaO_2_ = 99.5% × (1 – PULM-Shunt) + S_PA_O_2_ × PULM-Shunt (16)
